# Divergent Atrial Transcriptomic Signatures in Diabetic and Nondiabetic Atrial Fibrillation

**DOI:** 10.1002/joa3.70407

**Published:** 2026-07-17

**Authors:** Kenshi Yoshimura, Yuya Kiriake, Hiroki Osanai, Mengyan Wei, Shinichiro Kume, Tatsuki Kurokawa, Masaki Morishima, Ryusuke Suzuki, Shinji Miyamoto, Katsushige Ono

**Affiliations:** ^1^ Department of Pathophysiology Oita University School of Medicine Oita Japan; ^2^ Department of Cardiovascular Surgery Oita University School of Medicine Oita Japan; ^3^ Department of Food Science and Nutrition Kindai University Nara Japan; ^4^ Department of Cardiovascular Surgery Japanese Red Cross Kumamoto Hospital Kumamoto Japan; ^5^ Oita Shimogori Hospital Oita Japan

## Abstract

**Background:**

Diabetes mellitus (DM) increases the risk of atrial fibrillation (AF), but the molecular mechanisms underlying DM‐related atrial remodeling remain unclear. This study aimed to characterize transcriptomic differences, including microRNA (miRNA) profiles, between atrial tissue from AF patients with and without DM.

**Methods:**

Right atrial appendage samples were collected from 12 patients with AF undergoing cardiac surgery (six with DM and six without DM). Total RNA was analyzed by high‐throughput RNA sequencing. Differentially expressed genes (DEGs) and miRNAs were identified using Welch's *t*‐test (*p* < 0.05), and pathway analyses were performed using Gene Ontology (GO) and Gene Set Enrichment Analysis (GSEA).

**Results:**

Forty‐six protein‐coding genes and nine miRNAs were differentially expressed between the two groups. Upregulated genes in the DM group, including *MYH6* and *SLN*, were related to contractile and calcium‐handling functions, while downregulated genes, such as *ADAMTS4*, *CP*, and histone family members, were linked to extracellular matrix and chromatin regulation. GO and GSEA analyses revealed activation of mitochondrial ATP synthesis pathways and suppression of immune and inflammatory signaling. Additionally, distinct miRNA expression changes—such as upregulation of *miR‐3120* and downregulation of *miR‐4524B* and *miR‐6503*—suggested potential epigenetic regulation mechanisms.

**Conclusions:**

Transcriptomic profiling revealed that AF with DM is characterized by enhanced mitochondrial metabolism, suppressed immune pathways, and altered miRNA expression. These findings suggest that DM modifies the atrial substrate through metabolic and epigenetic mechanisms, providing novel insights into AF pathogenesis in diabetic patients.

## Graphical abstract

Diabetes alters the atrial transcriptome in atrial fibrillation, characterized by enhanced mitochondrial ATP synthesis, suppressed immune and inflammatory pathways, and distinct miRNA expression profiles, suggesting metabolic and epigenetic remodeling of the atrial substrate.

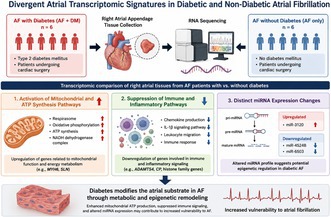

## 1 Introduction

Atrial fibrillation (AF) is the most common sustained cardiac arrhythmia encountered in clinical practice and a major contributor to cardiovascular morbidity and mortality worldwide. The pathophysiology of AF involves complex interactions among electrical, structural, and metabolic remodeling of the atrial myocardium. Among numerous comorbidities, diabetes mellitus (DM) is a well‐established risk factor for the development and persistence of AF. Epidemiological studies have shown that patients with DM have a 1.4–1.6‐fold higher risk of AF compared with those without DM, independent of age, hypertension, or coronary artery disease [1]. Several mechanisms have been proposed to explain this association. Chronic hyperglycemia promotes oxidative stress, accumulation of advanced glycation end products, and low‐grade inflammation, all of which contribute to atrial fibrosis and conduction abnormalities [2, 3, 4]. Diabetic hearts also exhibit mitochondrial dysfunction [5], altered calcium handling [6], and impaired energy metabolism [7], leading to electrical instability. These processes collectively create a pro‐arrhythmic atrial substrate. Animal studies have demonstrated that diabetic models show increased atrial fibrosis, activation of the MAPK and TGF‐β pathways, and changes in ion‐channel gene expression [8]. However, most of these data are derived from ventricular tissue or experimental models rather than from human atrial myocardium. Recent advances in high‐throughput RNA sequencing have enabled comprehensive profiling of gene expression, revealing the molecular pathways underlying AF‐related remodeling. Nevertheless, only a limited number of studies have compared the transcriptomic landscapes of AF patients with and without DM, and the molecular mechanisms that differentiate these two clinical entities remain largely unexplored. Furthermore, microRNAs (miRNAs)—key post‐transcriptional regulators implicated in diabetic cardiomyopathy—may also play distinct roles in modulating atrial remodeling in the diabetic state, but their contribution in the human atrial tissue has not been systematically characterized. Therefore, this study aimed to elucidate the molecular differences between diabetic and nondiabetic AF by performing comprehensive RNA sequencing of right atrial appendage tissue obtained from patients undergoing cardiac surgery. By integrating mRNA and miRNA expression profiles and performing gene ontology and pathway analyses, we sought to identify the transcriptomic signatures that distinguish diabetic AF from nondiabetic AF. These findings are expected to provide new insight into how diabetes modifies the atrial substrate and to suggest potential molecular targets for preventing or treating AF in diabetic patients.

## 2 Materials and Methods

### 2.1 Sample Collection

We enrolled 344 patients who underwent open‐heart surgery at Kumamoto Red Cross Hospital between August 2012 and January 2021. During surgery, the right atrial appendage was resected for venous drainage cannulation to establish extracorporeal circulation. The tissue was immediately placed in a tube filled with RNAlater solution (Invitrogen, USA), stored overnight at 4°C, and then transferred to a deep freezer until analysis. Among these patients, there were 18 patients who had AF. We selected six patients with atrial fibrillation and diabetes mellitus (DM group: *n* = 6) and six patients with atrial fibrillation without DM (non‐DM group: *n* = 6), as the male/female ratio and chronic/paroxysmal atrial fibrillation ratio were the same in both groups. Thus, among the 18 patients with atrial fibrillation, six patients with diabetes mellitus and six patients without diabetes mellitus were selected for RNA‐seq analysis based on sample availability, RNA quality, and balance of major clinical characteristics between groups, including sex and AF subtype (paroxysmal or chronic). Written informed consent was obtained from all patients. This study was approved by the Institutional Review Board of Kumamoto Red Cross Hospital (Approval No. 81–82).

### 2.2 mRNA Isolation

Total RNA was extracted using TRIzol Reagent (Thermo Fisher Scientific, MA, USA) according to the manufacturer's protocol. RNA quality was assessed using a Bioanalyzer (Agilent, CA, USA). All samples had RNA Integrity Numbers (RIN) greater than 7.0, which is recommended for RNA sequencing (RNA‐seq).

### 2.3 RNA‐Seq Data Acquisition

RNA‐seq libraries were prepared using the poly(A) selection method (NEBNext Poly(A) mRNA Magnetic Isolation Module, New England BioLabs, Ipswich, MA, USA) and a strand‐specific method (NEBNext Ultra II Directional RNA Library Prep Kit, New England BioLabs). Sequencing was performed on the Illumina NovaSeq 6000 platform (Illumina, San Diego, CA, USA). Read counts were normalized to transcripts per million (TPM), and comparisons between the DM and non‐DM groups were performed using TPM data.

### 2.4 Gene Expression Analysis

Gene expression levels between the DM and non‐DM groups were compared using log2(TPM + 1) values. Differences in expression were quantified as log2‐transformed fold changes, defined as the difference in mean log2(TPM + 1) values between groups. Welch's *t*‐test was applied, and *p*‐values < 0.05 were considered statistically significant.

### 2.5 Statistical Analysis

All statistical analyses were conducted using RStudio (version 2022.07.1) with R (version 4.2.1). Gene Ontology (GO) enrichment analyses were performed using the “clusterProfiler” package (version 4.4.4) from Bioconductor. Additional analyses were performed using Gene Set Enrichment Analysis (GSEA) software (version 4.2.3; https://www.gsea‐msigdb.org/gsea/index.jsp).

### 2.6 GO Enrichment Analysis

GO enrichment analysis was performed on differentially expressed genes with fold change ≥ 1.2 or ≤ 1/1.2 and *p*‐value ≤ 0.05 (Welch's *t*‐test). The fold‐change threshold was chosen to ensure that at least 100 genes were included. Cutoff values were set at adjusted *p*‐value ≤ 0.05 using the false discovery rate (FDR) method. A gene‐concept network was constructed to illustrate genes involved in each enriched GO term.

### 2.7 Gene Set Enrichment Analysis (GSEA)

All genes expressed in at least one sample were included in GSEA. Expression values were log2(TPM + 1). Ranking metrics were calculated using the “Signal2Noise” statistic, defined as:

$$\text{Signal}2\text{Noise}=\left(\mathrm{\mu A}-\mathrm{\mu B}\right)/\left(\mathrm{\sigma A}+\mathrm{\sigma B}\right)$$

where μA and μB are the means of groups A and B, respectively, and σA and σB are their standard deviations.

Permutation number was set to 1000, with “gene set” as the permutation type. Gene sets were obtained from the Gene Ontology database. Enrichment maps were created using the Enrichment Map app in Cytoscape (version 3.9.1). Default parameters were used (*p*‐value cutoff = 0.005, FDR *q*‐value cutoff = 0.1). The similarity metric was set to the combined Jaccard and Overlap coefficient (combined constant = 0.5, cutoff = 0.375).

### 2.8 Comparison With Animal Model Data

Because all human samples were from patients with atrial fibrillation, we compared our data with animal models to assess the effects of diabetes‐related cardiac remodeling. RNA‐seq data were obtained from the Gene Expression Omnibus (GSE161931 and GSE197999), representing spontaneous diabetic cardiomyopathy in db/db mice versus healthy controls, and streptozotocin‐induced diabetic cardiomyopathy in rats versus controls, respectively. All data were transformed to log_2_(TPM + 1) from raw count data extracted from each dataset and fold changes were calculated in the same way as described above. Human homologs of differentially expressed genes were identified using the “biomaRt” package from Bioconductor, and the overlap of differentially expressed genes between human and animal models was examined. For comparison with the rat model (GSE197999), fold‐change thresholds for up‐ and downregulation were set to 1.5 and 1/1.5, respectively. For the mouse model (GSE161931), thresholds were set to 1.2 and 1/1.2 due to the smaller number of intersecting genes. The fold‐change thresholds were decided as at least one overlapped gene could appear.

## 3 Results

### 3.1 Patient Characteristics

Table 1 summarizes the clinical characteristics of the DM and non‐DM patient groups. The mean ages were 72.8 ± 8.1 years in the DM group and 75.7 ± 5.9 years in the non‐DM group. The male‐to‐female ratio was 4:2 in both groups. No significant differences were observed in age, BMI, or body surface area between the groups. Hypertension was present in four of six patients in the DM group and in all patients in the non‐DM group, without statistical significance. HbA1c was significantly higher in the DM group than in the non‐DM group (7.2% ± 2.2% vs. 5.4% ± 0.3%, *p* < 0.001). Among electrolytes, serum potassium was significantly elevated in the DM group (4.9 ± 0.3 mEq/L vs. 4.4 ± 0.3 mEq/L, *p* = 0.04). No significant differences were detected in serum creatinine, sodium, chloride, heart rate, ejection fraction, or left atrial size. Two cases of paroxysmal AF were included in each group. No significant differences in medication use were identified between the groups.

**TABLE 1:** Patient characteristics.

	DM group (*n* = 6)	nonDM group (*n* = 6)	*p*
Age	72.8 ± 8.1	75.7 ± 5.9	0.54
Sex (male)	4	4	1.00
BMI	21.6 ± 2.5	20.2 ± 3.1	0.64
BSA	1.47 ± 0.2	1.46 ± 0.2	0.94
Procedures			
CABG	4	2	
Valve surgery	2	3	
Aorta surgery	0	1	
Hypertension	4	6	0.44
HbA1c (%)	7.1 ± 2.2	5.4 ± 0.3	0.0004*
BNP (pg/mL)	380.8 ± 335.6	266.7 ± 78.9	0.006*
Cr (mg/dL)	1.00 ± 0.23	0.77 ± 0.16	0.10
Na^+^ (mEq/L)	139.5 ± 3.2	139.5 ± 2.1	1.000
K^+^ (mEq/L)	4.9 ± 0.3	4.4 ± 0.3	0.04*
Cl^−^ (mEq/L)	103.7 ± 2.0	103.8 ± 2.9	0.92
HR (BPM)	74.5 ± 7.1	76.7 ± 16.5	0.79
Paroxysmal AF	2	2	1.00
LVEF (%)	49.3 ± 17.3	52.8 ± 12.9	0.72
LAD (mm)	46.3 + 5.6	44.9 ± 6.5	0.73
Moderate or severe TR	1	2	1.00
Medication			
β blocker	4	4	1.00
Statin	5	4	1.00
Ca‐antagonist	2	1	1.00
ACE, ARB	3	5	0.54
Antiarrythmics	2	1	1.00

### 3.2 Gene Expression Comparison

**FIGURE 1:**
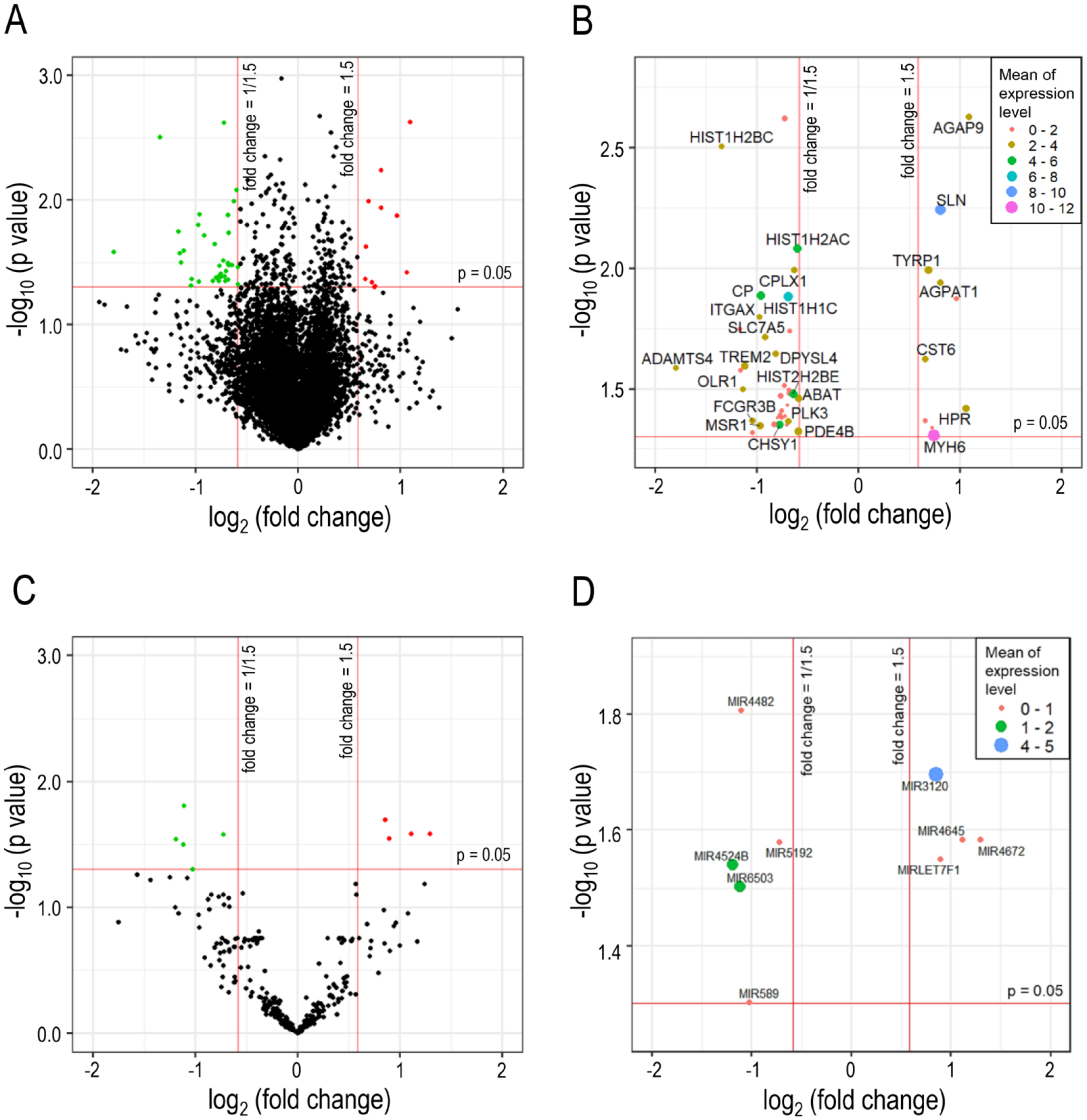
Volcano plot of all expressed protein‐coding genes in the right atrial appendage obtained from RNA sequencing, where the x‐axis denotes log2 fold change and the y‐axis denotes –log10 *p*‐value. Vertical red lines represent fold changes of 1/1.5 and 1.5, and the horizontal red line represents *p* = 0.05. Red and green dots indicate up‐ and downregulated genes, respectively. (B) Volcano plot of protein‐coding genes with fold change < 1/1.5 or > 1.5 and *p* < 0.05. Dot size represents mean expression calculated as log_2_(TPM + 1). (C) Volcano plot of all expressed microRNAs. Vertical and horizontal red lines are as in (A). Red dots represent upregulated and green dots represent downregulated microRNAs. (D) Volcano plot of microRNAs with fold change < 1/1.5 or > 1.5 and *p* < 0.05. Dot size represents mean expression calculated as log_2_(TPM + 1).

RNA‐seq data were analyzed, and transcripts per million (TPM)–normalized values were compared between groups. Figure 1 presents the volcano plots of differential expression. Figure 1A shows protein‐coding genes, whereas Figure 1C shows microRNAs (miRNAs). Figure 1B,D display genes that met significance criteria (fold change > 1.5 or < 1/1.5, *p* < 0.05), with plot size and color indicating expression level. Table 2 lists upregulated and downregulated protein‐coding genes, ordered by log2 fold change. Eleven genes were upregulated and 35 were downregulated. The most highly expressed upregulated genes were *MYH6*, *SLN*, and *TYRP1*, whereas the most highly expressed downregulated genes were *H1‐2*, *H2BC21*, and *CP*. Table 3 lists upregulated and downregulated miRNAs. Four upregulated and five downregulated miRNAs were identified. *miR3120* exhibited the smallest log_2_ fold change among upregulated miRNAs but the highest expression level. Among downregulated miRNAs, *miR4524B* and *miR6503* demonstrated both small fold changes and high expression levels.

**TABLE 2:** Differentially expressed genes in DM group.

Up‐regulated genes				
Gene	log_2_FC	*p*	Mean of expression level	Protein
AGAP9	1.092	0.002	2.467	Arf‐GAP with GTPase, ANK repeat and PH domain‐containing protein 9
HPR	1.059	0.038	3.450	Haptoglobin‐related protein
PCSK2	0.967	0.013	1.593	Proprotein convertase subtilisin/kexin type 2, Neuroendocrine convertase 2
SLN	0.810	0.006	8.281	Sarcolipin
AGPAT1	0.809	0.011	2.571	1‐acylglycerol‐3‐phosphate O‐acyltransferase 1
CABCOCO1	0.753	0.049	0.556	Ciliary‐associated calcium‐binding coiled‐coil protein 1
MYH6	0.745	0.050	11.676	Myosin heavy chain 6
CAPN6	0.723	0.046	0.600	Calpain‐6
TYRP1	0.691	0.010	3.829	Tyrosinase related protein 1
CST6	0.661	0.024	2.358	Cystatin E/M
TMEM163	0.656	0.043	1.507	Transmembrane protein 163

Down‐regulated genes				
Gene	log_2_FC	*p*	Mean of expression level	Protein
ADAMTS4	−1.798	0.026	2.319	A disintegrin and metalloproteinase with thrombospondin motifs 4
H2BC4	−1.166	0.018	1.331	Histone H2B type 1‐C/E/F/G/I
H2BC18	−1.166	0.018	1.331	Histone H2B type 2‐F
SLC11A1	−1.155	0.027	1.551	Solute carrier family 11 member 1, Natural resistance‐associated macrophage protein 1
OLR1	−1.140	0.032	2.142	Oxidized low‐density lipoprotein receptor 1
TREM2	−1.118	0.025	3.820	Triggering receptor expressed on myeloid cells 2
H2BC8	−1.043	0.048	1.715	Histone H2B type 1‐C/E/F/G/I
FCGR3B	−1.040	0.043	2.403	Low AFibfinity immunoglobulin gammAFibc region receptor III‐B
ITGAX	−0.973	0.016	2.035	Integrin alpha‐X
MSR1	−0.965	0.045	3.690	Macrophage scavenger receptor types I and II
CP	−0.963	0.013	4.926	Ceruloplasmin
SLC7A5	−0.915	0.019	2.466	Solute carrier family 7 member 5, Large neutral amino acids transporter small subunit 1
MME	−0.831	0.044	1.791	Membrane metalloendopeptidas, neprilysin
DPYSL4	−0.813	0.023	2.508	Dihydropyrimidinase like 4
IFNG	−0.797	0.042	0.598	Interferon gamma
CHSY1	−0.772	0.045	4.617	Chondroitin sulfate synthase 1
MCF2	−0.768	0.040	1.309	MCF.2 cell line derived transforming sequence, Proto‐oncogene DBL
AP2A2	−0.767	0.034	1.796	Adaptor related protein complex 2 subunit alpha 2
NOCT	−0.751	0.042	1.686	Nocturnin
ZMYND15	−0.751	0.039	1.692	Zinc finger MYND domain‐containing protein 15
SH2D2A	−0.727	0.031	1.417	SH2 domain containing 2A
ACER2	−0.723	0.002	1.970	Alkaline ceramidase 2
FFAR2	−0.717	0.041	0.957	Free fatty acid receptor 2
GPR84	−0.707	0.045	0.686	G protein‐coupled receptor 84
PROK2	−0.700	0.037	0.673	Prokineticin‐2
PLK3	−0.685	0.043	2.941	Polo like kinase 3
H1‐2	−0.684	0.013	6.169	H1.2 linker histone, cluster member
FAM163B	−0.680	0.032	1.693	Family with sequence similarity 163 member B
TNFRSF10C	−0.677	0.033	1.550	TNF receptor superfamily member 10c
MS4A14	−0.675	0.018	1.359	Membrane‐spanning 4‐domains subfamily A member 14
H2BC21	−0.643	0.033	5.271	H2B clustered histone 21
CPLX1	−0.627	0.010	2.841	Complexin‐1
H2AC6	−0.599	0.008	4.751	H2A clustered histone 6
ABAT	−0.588	0.035	3.348	4‐aminobutyrate aminotransferase
PDE4B	−0.586	0.048	3.847	cAMP‐specific 3′,5′‐cyclic phosphodiesterase 4B

**TABLE 3:** Differentially expressed miRNA in DM group.

Up‐regulated miRNA			
miRNA	log_2_FC	*p*	Mean of expression level
MIR4672	1.294	0.026	0.647
MIR4645	1.111	0.026	0.791
MIRLET7F1	0.894	0.028	0.447
MIR3120	0.853	0.020	4.140

Down‐regulated miRNA			
miRNA	log_2_FC	*p*	Mean of expression level
MIR4524B	−1.192	0.029	1.043
MIR6503	−1.120	0.031	1.271
MIR4482	−1.109	0.016	0.748
MIR589	−1.028	0.050	0.716
MIR5192	−0.729	0.026	0.365

### 3.3 GO Enrichment Analysis and Concept Network

**FIGURE 2:**
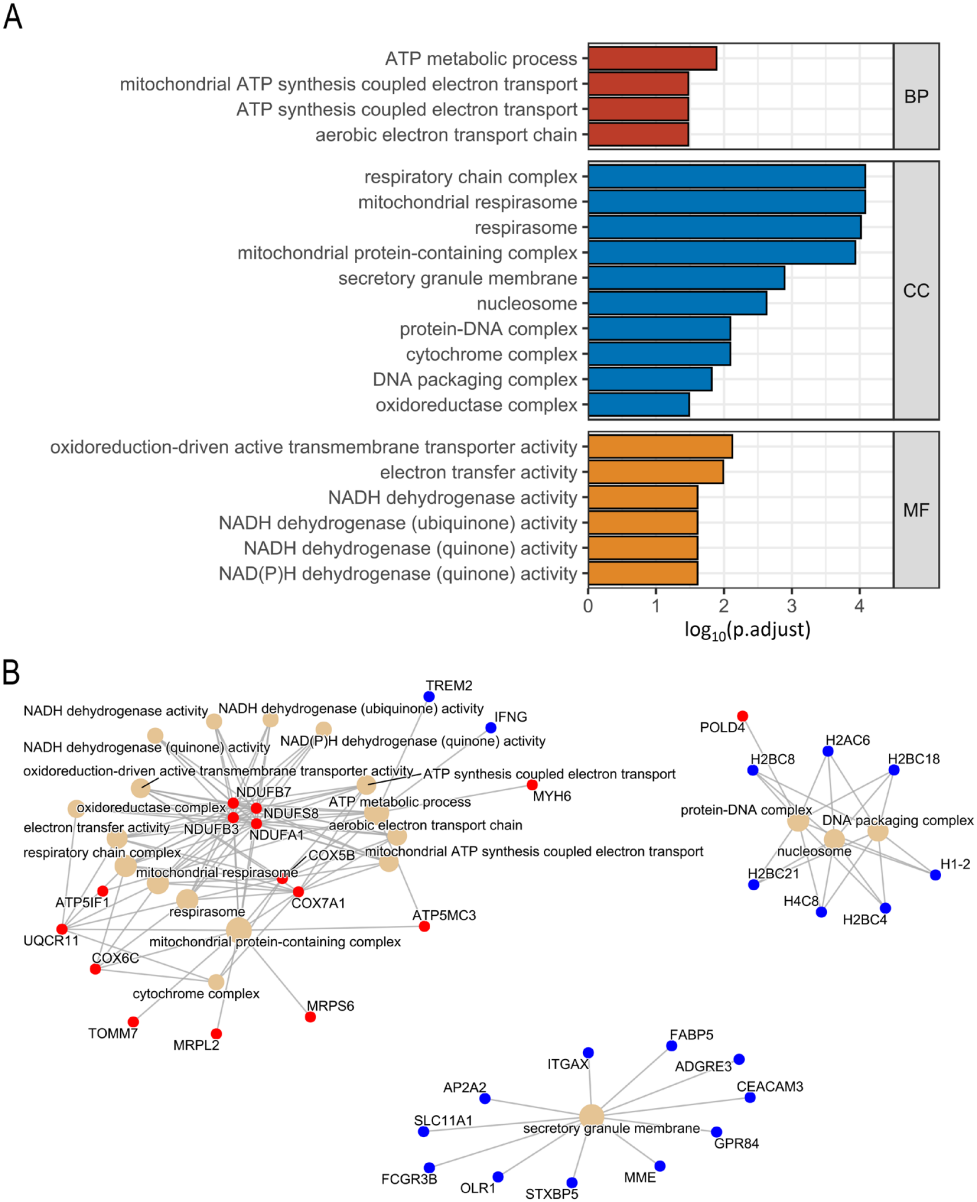
Top 20 results of Gene Ontology (GO) enrichment analysis for differentially expressed genes selected with fold change thresholds of 1/1.2 and 1.2 and *p* < 0.05. (B) Gene‐concept network of the top 20 GO terms and their associated genes. Red and blue small nodes represent up‐ and downregulated genes, respectively, while large orange nodes represent GO terms. Node size reflects the number of linked genes. BP, Biological process; CC, Cellular component; MF, Molecular function.

A total of 153 protein‐coding genes were selected for GO enrichment analysis using a threshold of fold change > 1.2 or < 1/1.2 with *p* < 0.05. The results are shown in Figure 2A. In the biological process category, GO terms associated with adenosine triphosphate (ATP) synthesis were enriched. In the cellular component category, terms related to mitochondrial respiratory complexes, nucleosomes, and the secretory granule membrane were identified. In the molecular function category, terms associated with ATP generation via the mitochondrial electron transport chain were enriched. A gene concept network was constructed (Figure 2B). Three principal clusters were identified: a mitochondrial ATP generation–related cluster, a nucleosome‐related cluster, and a secretory granule membrane–related cluster.

### 3.4 Gene Set Enrichment Analysis (GSEA)

**FIGURE 3:**
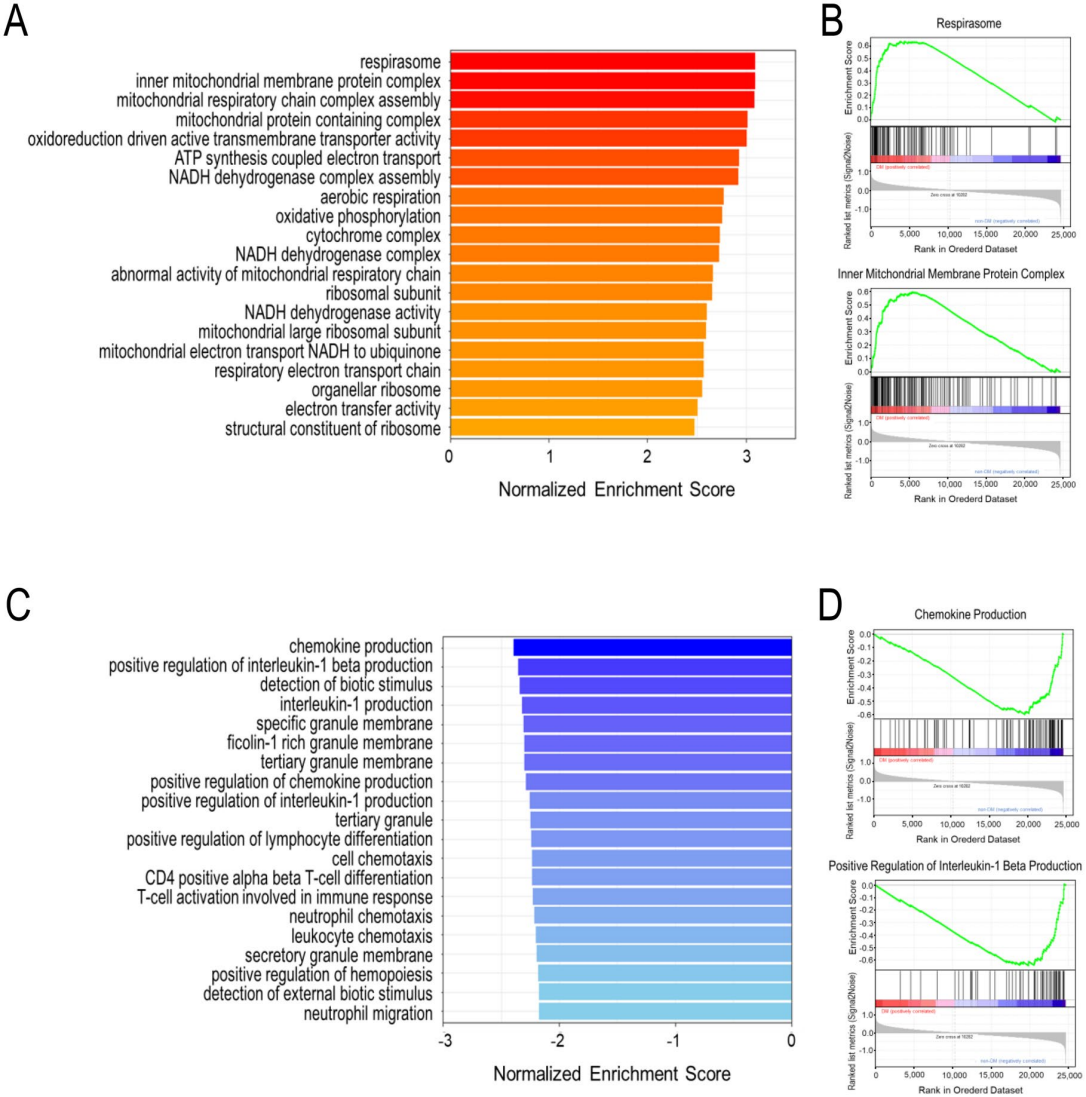
Top 20 upregulated (A) and down regulated gene sets (C) identified by Gene Set Enrichment Analysis (GSEA), ranked by normalized enrichment score (NES). Representative enrichment plots of the first and second most significantly upregulated (B) and downregulated gene sets (D). The top panel (green line) shows the running enrichment score. The middle panel (black ticks) indicates the positions of gene set members in the ranked gene list, where the red side represents top‐ranked and the blue side bottom‐ranked genes in the DM group. The bottom panel (gray bars) shows the ranking metric values (“signal‐to‐noise”).

**FIGURE 4:**
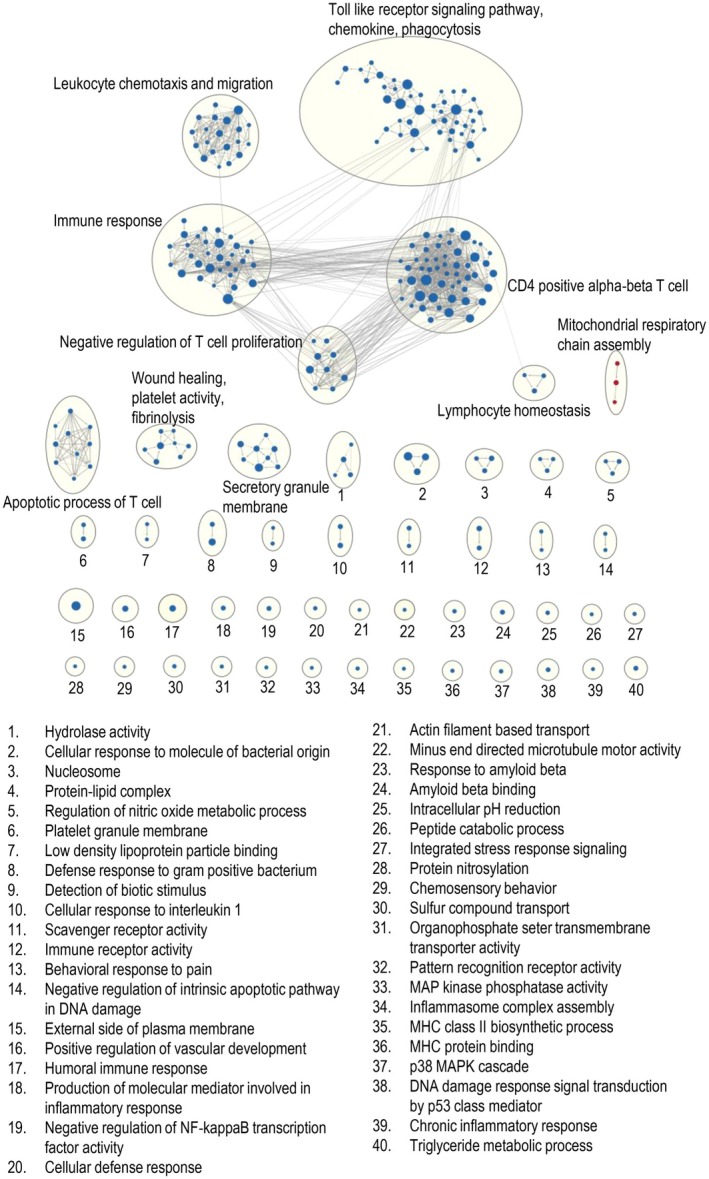
Enrichment map generated from GSEA results. Nodes represent gene sets, and edges indicate shared genes between nodes. Clusters of related gene sets were generated using the AutoAnnotate app. Cluster names were refined with reference to the GO graph from the Gene Ontology website (https://www.geneontology.org/).

Figure 3 present the results of GSEA based on the GO database. Figure 3A,C display normalized enrichment scores for upregulated and downregulated gene sets, respectively. Figure 3B,D highlight the top two gene sets in each category. Upregulated gene sets included “respirasome” and “inner mitochondrial membrane protein complex,” both associated with ATP generation at the mitochondrial membrane, consistent with the GO enrichment analysis. Downregulated gene sets included “secretory granule membrane,” “specific granule membrane,” “ficolin‐1–rich granule membrane,” and “tertiary granule membrane.” The leading downregulated gene sets—“chemokine production” and “positive regulation of interleukin‐1 beta production”—along with others, were predominantly associated with inflammatory and immune responses. The enrichment map (Figure 4) demonstrates the interrelationships among GO terms. Each node represents a GO term. Most nodes were downregulated (blue). The largest networks involved immune response, leukocyte migration, and leukocyte apoptosis. Additional networks related to platelet activation, coagulation, and fibrinolysis were also observed. Only three upregulated nodes (red), all related to mitochondrial function, were identified.

### 3.5 Comparison With Animal Model Data

**FIGURE 5:**
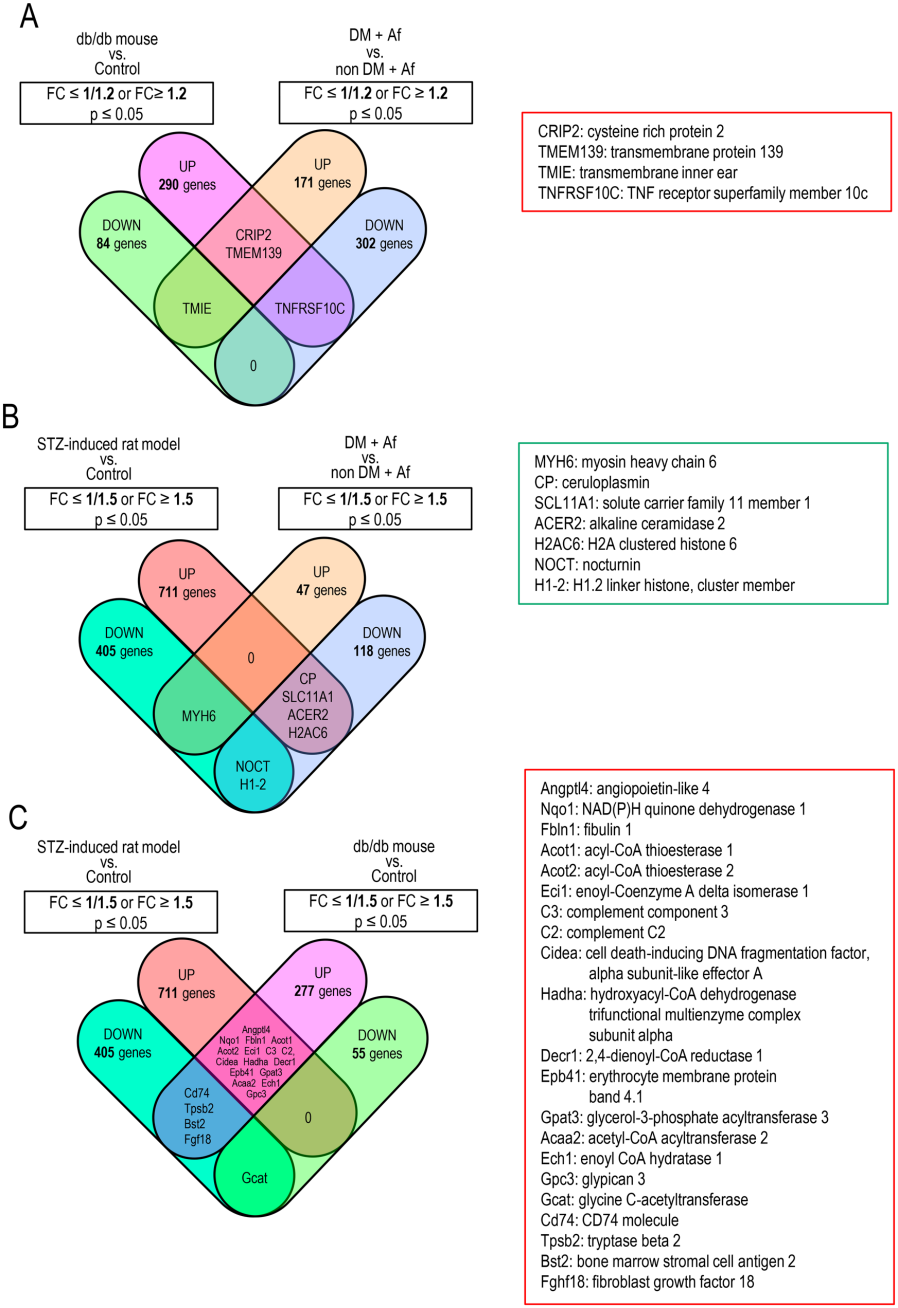
(A, B) Comparison of our data with animal experimental data from the Gene Expression Omnibus (GSE161931 and GSE197999). For the mouse model (GSE161931: Db/db vs. control), fold‐change thresholds were 1/1.2 and 1.2 with *p* < 0.05. For the rat model (GSE197999: STZ‐induced vs. control), thresholds were 1/1.5 and 1.5 with *p* < 0.05. (C) Comparison of gene expression between mouse (GSE161931) and rat (GSE197999) models, with thresholds of 1/1.2 and 1.2 and *p* < 0.05 for both. Gene symbols: ACAA2, acetyl‐CoA acyltransferase 2; ACER2, alkaline ceramidase 2; ACOT1, acyl‐CoA thioesterase 1; ACOT2, acyl‐CoA thioesterase 2; ANGPTL4, angiopoietin‐like 4; BST2, bone marrow stromal cell antigen 2; C2, complement C2; C3, complement component 3; CD74, CD74 molecule; CIDEA, cell death‐inducing DNA fragmentation factor, alpha subunit‐like effector A; CP, ceruloplasmin; CRIP2, cysteine rich protein 2; DECR1, 2,4‐dienoyl‐CoA reductase 1; ECH1, enoyl CoA hydratase 1; ECI1, enoyl‐Coenzyme A delta isomerase 1; EPB41, erythrocyte membrane protein band 4.1; FBLN1, fibulin 1; FGF18, fibroblast growth factor 18; GCAT, glycine C‐acetyltransferase; GPAT3, glycerol‐3‐phosphate acyltransferase 3; GPC3, glypican 3; H1‐2, H1.2 linker histone, cluster member; H2AC6, H2A clustered histone 6; HADHA, hydroxyacyl‐CoA dehydrogenase trifunctional multienzyme complex subunit alpha; MYH6, myosin heavy chain 6; NOCT, nocturnin; NQO1, NAD(P)H quinone dehydrogenase 1; SCL11A1, solute carrier family 11 member 1; TMEM139, transmembrane protein 139; TMIE, transmembrane inner ear; TNFRSF10C, TNF receptor superfamily member 10c; TPSB2, tryptase beta 2.

Figure 5 compares the present dataset with publicly available animal model datasets (GSE161931 and GSE197999) from the Gene Expression Omnibus (GEO). In the db/db mouse model, *CRIP2* (*Chip2*) and *TMEM139* (*Tmem139*) were commonly upregulated in both the model and our samples. *TMIE* (*Tmie*) was upregulated in our samples but downregulated in the mouse model, whereas *TNFRSF10C* (*Tnfrsf10c*) was upregulated in the mouse model but downregulated in our samples. No commonly downregulated genes were observed. In the STZ‐induced rat model, no commonly upregulated genes were identified. *NOCT* (*Noct*) and *H1‐2* (*H1‐2*) were commonly downregulated in both datasets. *MYH6* (*Myh6*) was upregulated in our samples but downregulated in the rat model. In contrast, *CP* (*Cp*), *SLC11A1* (*Slc11a1*), and *ACER2* (*Acer2*) were downregulated in our samples but upregulated in the rat model. These discrepancies may be attributable to tissue specificity, as all samples in the present study were obtained from atrial tissue, whereas the GEO datasets were derived from ventricular tissue in mice or rats.

## 4 Discussion

AF is one of the most common arrhythmias encountered in clinical practice and is a complex polygenic disorder in which various factors—including its precipitating triggers, underlying conditions, the types of atrial myocardial remodeling, and the genetic diversity of expressed genes—interact intricately to contribute to its pathogenesis [9, 10, 11]. Established risk factors include obesity, physical inactivity, obstructive sleep apnea, hypertension, coronary artery disease, and heart failure. DM is also recognized as a significant contributor to AF incidence [12, 13, 14]. In DM patients, the heart exhibits increased left ventricular mass and wall thickness, along with augmented pulse pressure and cardiac output, which chronically stress the left atrium and contribute to atrial enlargement and remodeling [15]. Moreover, accumulation of advanced glycation end products promotes atrial fibrosis, thereby facilitating AF development [16, 17]. In this study, transcriptomic profiling of atrial tissue from AF patients with and without DM identified 11 upregulated and 35 downregulated protein‐coding genes in the DM group (fold change > 1.5 or < 1/1.5, *p* < 0.05). Among the upregulated genes, MYH6, encoding myosin heavy chain alpha, displayed the highest average expression. While MYH6 has been proposed as a potential biomarker for diabetes [18], previous studies reported downregulation in ventricular cardiomyocytes in Type 2 diabetic animal models [19] and in STZ‐induced rats (GSE197999). This discrepancy may reflect tissue‐specific expression, as MYH6 is more abundantly expressed in atrial than ventricular myocardium [20], highlighting its potential importance in atrial pathophysiology in DM. SLN, encoding sarcolipin, another gene with altered expression, regulates sarcoplasmic/endoplasmic reticulum Ca^2+^ ATPase 2a (SERCA2a)‐mediated calcium reuptake into the sarcoplasmic reticulum. Prior studies demonstrated increased SLN expression in diabetic ventricular tissue correlating with HbA1c and impaired left ventricular function [21]. In our study, SLN expression was also elevated in atrial tissue, suggesting a common response in both atrial and ventricular myocardium. Notably, SLN is typically suppressed in AF atria, which may enhance sarcoplasmic reticulum Ca^2+^ reuptake, contributing to AF‐associated remodeling [22, 23]. Despite this, DM appeared to partially restore SLN expression relative to non‐DM AF patients, warranting further investigation into its role in atrial calcium cycling under diabetic conditions. ADAMTS4, the most downregulated gene in our cohort, encodes a disintegrin and metalloproteinase with thrombospondin motif 4 implicated in cartilage remodeling. Although its cardiac role in DM has not been previously reported, we observed reduced expression in atrial tissue from DM patients, suggesting novel pathophysiological relevance. CP, encoding ceruloplasmin, was also suppressed. Ceruloplasmin exhibits both antioxidant and prooxidant activity, and its modulation in the diabetic atrium may influence oxidative stress, although its specific cardiac implications remain unclear. Mitochondrial dysfunction is a hallmark of diabetic myocardium, often resulting from hyperglycemia‐induced NADH/NAD^+^ imbalance and dysregulated energy metabolism [24]. GO enrichment analysis revealed upregulation of genes associated with mitochondrial ATP synthesis, oxidoreductase complexes, and NADH dehydrogenase, suggesting enhanced complex I activity in atrial tissue. Whether these changes represent direct consequences of DM or compensatory mechanisms remains to be elucidated. Histone gene expression was also affected, with downregulation of core histones H2A and H2B, and the linker histone H1–2. Such alterations may reflect hyperglycemia‐mediated epigenetic changes, as advanced glycation and oxidative stress can impair histone function [25]. GSEA further indicated suppression of immune‐related gene sets, consistent with observed histone alterations [3, 26]. Given the higher proportion of immune cells in atrial versus ventricular tissue [27], these changes may significantly influence atrial pathophysiology in DM. Previous animal studies demonstrated increased atrial IL‐6 and IL‐1β and enhanced AF susceptibility in diabetic mice, mediated by MAPK10 signaling [28]. Anti‐diabetic medications, including metformin, GLP‐1 receptor agonists, and DPP‐4 inhibitors, reduce myocardial inflammation [29, 30, 31, 32]. In our cohort, no significant differences in IL‐6, IL‐1β, or MAPK10 were observed between DM and non‐DM AF patients, likely reflecting glycemic control and medication effects, as well as confounding comorbidities. Neither GO enrichment nor GSEA revealed significant alterations in coagulation pathways, consistent with our analysis of right atrial appendage tissue, which is less prone to thrombus formation compared with the left atrium. Comparison with animal models revealed limited overlap, likely due to differences in tissue type and absence of AF in experimental animals.

Although the present study identified nine distinct microRNAs (four upregulated and five downregulated) that may characterize the molecular signature of DM‐associated AF, the functional significance of these microRNAs remains to be fully elucidated, even after comparison with previously published datasets. MicroRNAs have been extensively investigated in the context of diabetic cardiomyopathy in both humans and animal models [33, 34]. However, the majority of these studies have focused primarily on ventricular tissue or cardiomyocytes, rather than on atrial myocardium. A limited number of investigations have reported alterations in several microRNAs (including miR‐532‐3p, miR‐34a, miR‐208a, and miR‐21) in atrial tissue under diabetic conditions [35, 36, 37, 38]; nevertheless, these microRNAs were not detected in the present analysis. Direct comparative studies using human atrial tissue to delineate molecular differences between DM‐associated and non‐DM‐associated AF are exceedingly limited, largely due to the difficulty in obtaining clinical atrial specimens—particularly from the left or right atrial appendage—and the consequent small sample sizes. As a result, few investigations possess adequate statistical power to stratify AF patients by diabetic status. Most available data derive from studies comparing (A) AF versus sinus rhythm or (B) DM versus non‐DM myocardium in general. This paucity of direct comparative evidence has been consistently highlighted in recent reviews and meta‐analyses [39]. Further comprehensive investigations employing well‐characterized human atrial tissue cohorts are clearly warranted to elucidate these unresolved issues.

This study has several limitations. Sinus rhythm atrial tissue was not analyzed, preventing assessment of AF‐specific changes. Patient backgrounds, comorbidities, and prior surgical interventions were heterogeneous. Additionally, animal models predominantly involve ventricular tissue, limiting direct cross‐species comparisons.

In conclusion, this study provides the first comprehensive characterization of gene expression profiles in atrial tissue from diabetic AF patients. Differentially expressed genes, including *MYH6*, *SLN*, *ADAMTS4*, *CP*, and histones, suggest multiple mechanisms through which DM may modulate atrial remodeling and function. Although the opposite regulation of MYH6 compared with previous ventricular studies may reflect tissue‐specific differences between atrial and ventricular myocardium, this interpretation remains speculative because ventricular tissue was not analyzed in the present study. Furthermore, atrial fibrillation itself may influence MYH6 expression through atrial remodeling processes independent of diabetes mellitus. Therefore, the observed MYH6 upregulation should be interpreted cautiously, and further studies directly comparing atrial and ventricular tissues under diabetic and nondiabetic conditions are warranted. Although differential expression analysis was performed using TPM‐normalized expression values and Welch's *t*‐test rather than count‐based statistical frameworks such as DESeq2 or edgeR, the present study was designed primarily as an exploratory and hypothesis‐generating investigation using a limited number of surgically obtained human atrial tissue samples. Because of the substantial biological heterogeneity and small sample size inherent to human atrial appendage specimens, particularly in cohorts stratified by both atrial fibrillation and diabetes status, stringent multiple‐testing correction may markedly reduce sensitivity to biologically relevant transcriptomic changes. Therefore, we focused not only on individual differentially expressed genes but also on the consistency of pathway‐level findings across GO enrichment analysis, GSEA, and network analyses. Nevertheless, the identified transcriptomic signatures should be interpreted cautiously, and further validation using larger cohorts and count‐based RNA‐seq analytical methods with appropriate multiple‐testing correction will be necessary to confirm the robustness and reproducibility of these findings. We also note the methodological limitations. Because the RNA‐seq libraries were prepared using a poly(A) selection‐based protocol optimized for mRNA analysis rather than dedicated small RNA sequencing, the miRNA expression results should be interpreted as exploratory findings. Further validation using small RNA‐seq or quantitative PCR approaches will be necessary to confirm these observations. In addition, the cross‐species comparison using publicly available GEO datasets should be interpreted cautiously. The human, mouse, and rat datasets were generated from independent studies using different sequencing platforms, library preparation methods, and experimental conditions, which may introduce batch effects and technical variability. Furthermore, the animal datasets were derived primarily from ventricular tissue, whereas the present study analyzed human atrial tissue, potentially contributing to tissue‐specific transcriptomic differences. Species‐specific biological variation between humans and rodents may also limit direct comparability. Although all datasets were transformed using the same log2(TPM + 1) approach before comparison, the datasets were not uniformly reprocessed from raw sequencing files using an identical bioinformatics pipeline. Therefore, the overlap analysis should be considered exploratory rather than definitive. Because of the relatively small sample size and the exploratory design of the present study, differential expression analysis using unadjusted *p*‐values may include false‐positive findings. Therefore, the identified transcriptomic alterations should be interpreted cautiously and require validation in larger independent cohorts using more stringent statistical correction methods. Further functional analyses are warranted to clarify their roles in AF pathophysiology under diabetic conditions.

## Funding

The authors have nothing to report.

## Ethics Statement

Informed consent was obtained from the patient for participation and publication of this study. This study was approved by the Institutional Review Board of Kumamoto Red Cross Hospital (Approval No. 81‐2).

## Conflicts of Interest

The authors declare no conflicts of interest.

## Data Availability

The data that support the findings of this study are available on request from the corresponding author. The data are not publicly available due to privacy or ethical restrictions.
